# Cortical Proprioceptive Processing Is Altered by Aging

**DOI:** 10.3389/fnagi.2018.00147

**Published:** 2018-06-14

**Authors:** Harri Piitulainen, Santtu Seipäjärvi, Janne Avela, Tiina Parviainen, Simon Walker

**Affiliations:** ^1^Sensorimotor Systems Group, Department of Neuroscience and Biomedical Engineering, Aalto University School of Science, Espoo, Finland; ^2^Biology of Physical Activity and Neuromuscular Research Center, Faculty of Sport and Health Sciences, University of Jyväskylä, Jyväskylä, Finland; ^3^Centre for Interdisciplinary Brain Research, Department of Psychology, University of Jyväskylä, Jyväskylä, Finland

**Keywords:** proprioception, sensorimotor cortex, aging, somatosensory, passive movement, balance, coherence, sensorimotor integration

## Abstract

Proprioceptive perception is impaired with aging, but little is known about aging-related deterioration of proprioception at the cortical level. Corticokinematic coherence (CKC) between limb kinematic and magnetoencephalographic (MEG) signals reflects cortical processing of proprioceptive afference. We, thus, compared CKC strength to ankle movements between younger and older subjects, and examined whether CKC predicts postural stability. Fifteen younger (range 18–31 years) and eight older (66–73 years) sedentary volunteers were seated in MEG, while their right and left ankle joints were moved separately at 2 Hz (for 4 min each) using a novel MEG-compatible ankle-movement actuator. Coherence was computed between foot acceleration and MEG signals. CKC strength at the movement frequency (F0) and its first harmonic (F1) was quantified. In addition, postural sway was quantified during standing eyes-open and eyes-closed tasks to estimate motor performance. CKC peaked in the gradiometers over the vertex, and was significantly stronger (~76%) at F0 for the older than younger subjects. At F1, only the dominant-leg CKC was significantly stronger (~15%) for the older than younger subjects. In addition, CKC (at F1) was significantly stronger in the non-dominant than dominant leg, but only in the younger subjects. Postural sway was significantly (~64%) higher in the older than younger subjects when standing with eyes closed. Regression models indicated that CKC strength at F1 in the dominant leg and age were the only significant predictors for postural sway. Our results indicated that aging-related cortical-proprioceptive processing is altered by aging. Stronger CKC may reflect poorer cortical proprioceptive processing, and not solely the amount of proprioceptive afference as suggested earlier. In combination with ankle-movement actuator, CKC can be efficiently used to unravel proprioception-related-neuronal mechanisms and the related plastic changes in aging, rehabilitation, motor-skill acquisition, motor disorders etc.

## Introduction

The human neuromuscular system is strongly affected by aging (Larsson et al., 1979; Shaffer and Harrison, 2007; Mitchell et al., 2012; Rowan et al., 2012; Kulmala et al., 2014; Frontera, 2017). Aging-related deterioration of the motor system is demonstrated as a decline in motor performance such as loss of maximum muscle force (Frontera et al., 1991; Lindle et al., 1997), less accurate bimanual coordination (Maes et al., 2017), increased postural sway during static standing (Hasselkus and Shambes, 1975; Era and Heikkinen, 1985; Baloh et al., 1994), worsening dynamic balance control (Piirainen et al., 2013), and alterations in cortical control of upright stance (Ozdemir et al., 2018). Postural instability is related to the risk of falling (Maki et al., 1994; Pajala et al., 2008) and the number of balance loss events during perturbation of the standing posture (Boisgontier et al., 2017). Motor control relies on vast sensory input from the environment (vision, audition, touch) and especially on the internal state of the motor system through proprioception. This internal model of motor system state and thus proprioceptive control of movement are somewhat altered by aging (Boisgontier and Nougier, 2013). The proprioceptors are located in muscles and joints, and sense limb positions, movements and forces (Proske and Gandevia, 2012). Impaired proprioception has been considered as a main contributor to balance loss in older adults (Lord and Ward, 1994; McChesney and Woollacott, 2000). Proprioceptive perception (joint position sense) is impaired with aging (Skinner et al., 1984; Kaplan et al., 1985; Goble et al., 2009), and is termed as “presbypropria” (Boisgontier et al., 2012). Little is known about aging-related deterioration of proprioception at the cortical level, but brain responses to proprioceptive stimulation have shown significant associations to postural control (Goble et al., 2011) and proprioceptive perception (Goble et al., 2012).

Corticokinematic coherence (CKC) quantifies the coupling between cortical activity, measured with magnetoencephalo- graphy (MEG) or electroencephalography (EEG) and limb kinematics (e.g., acceleration) during repetitive rhythmic active (Jerbi et al., 2007; Bourguignon et al., 2011) and passive (Piitulainen et al., 2013a, 2015; Smeds et al., 2017) movements. CKC peaks at the movement frequency and its harmonics, and it can be measured using various peripheral movement-related signals and motor tasks (Piitulainen et al., 2013b). CKC can be used for non-invasive functional cortex mapping to identify the human primary sensorimotor (SM1) cortex (Bourguignon et al., 2011, 2013). CKC primarily reflects proprioceptive processing in the SM1 cortex with negligible effect of cutaneous afference (Piitulainen et al., 2013a; Bourguignon et al., 2015). CKC corresponds to the timing of the strongest deflection of the cortical passive-movement-evoked field with an apparent latency of 50–100 ms (Piitulainen et al., 2015). However, it is not known how the strength of CKC is related with the efficiency of the proprioceptive processing, and whether the level of CKC is associated with motor performance. This relationship could be presumed if the motor tasks rely primarily on proprioception, e.g., when performing a balance task with eyes closed.

As CKC reflects cortical proprioceptive processing it could potentially be used to unravel proprioception-related-neuronal mechanisms both in healthy and clinical populations. Previous MEG studies, where passive movements have been generated by pneumatic cylinders (Alary et al., 2002) or pneumatic artificial muscles (Piitulainen et al., 2015), have focused on small-scale finger or toe movements only. In order to test for the associations between CKC and functional performance, such as locomotion or balance, CKC should be applied on lower limbs at the functional range of joint rotations. Here, we introduce a novel MEG-compatible ankle-movement actuator, and use it to quantify CKC during continuous dorsi-plantarflexion movement in a similar manner as done previously for hand, finger and hallux movements (Bourguignon et al., 2011; Piitulainen et al., 2013a, 2015). The movement actuator is nonmagnetic, and is therefore both MEG- and functional magnetic resonance imaging (fMRI) compatible. DC-motor-based movement actuators have been applied with EEG recordings (Desmedt and Ozaki, 1991; Mima et al., 1996; Ramos-Murguialday et al., 2012), but unfortunately DC-motors are not MEG-compatible.

Our primary aim was to study the effect of aging on CKC strength evoked by passive movements of dominant and non-dominant leg. We also aimed to study whether CKC predicts postural stability in a task relying on both vision and proprioception (standing eyes-open) or on proprioception alone (standing eyes-closed). We hypothesized that older subjects will show weaker CKC than the younger subjects, indicating impaired cortical proprioceptive processing. We also hypothesized that weaker CKC strength (i.e., worse cortical proprioceptive processing) would be reflected by higher postural sway during eyes-closed standing (Baloh et al., 1994), and thus CKC strength would be a significant predictor of the degree of postural sway. It is important to note that habitual physical activity levels, body mass and height were matched between the groups, and thus our results emphasize age-related effects.

## Materials and Methods

### Subjects

Fifteen younger (mean age 24.8 years, range 18–31 years, height 171 ± 11 cm, body mass 69 ± 12 kg; four males) and eight older sedentary volunteers (mean age 69.3 years, range 66–73 years, height 167 ± 9 cm, body mass 73 ± 15 kg; five males) were studied. They did not report any history of movement disorders or neurological disease, and were not taking medication affecting the central nervous system. The subjects were pre-screened to confirm that there were no differences in the level of physical activity performed between groups (i.e., all subjects habitually performed less than the recommended 150 min aerobic exercise per week and none were engaged in strength training). The subjects were specifically asked to declare the type of aerobic exercise e.g., walking or cycling et cetera (performed for at least 20 min of duration) and the number of times per week that they engaged in these aerobic exercises. The same questions were used for strength training. Subjects were blind to the eligibility criteria of the study so as not to influence their pre-study reporting. Average weekly aerobic exercise duration in younger (128 ± 76 min) was similar to those of older (124 ± 57 min). Their dominant leg was defined as the preferred leg to kick a ball (right leg in all subjects). This study was carried out in accordance with the recommendations of the Declaration of Helsinki, and the ethics committee of the University of Jyväskylä. The protocol was approved by the ethics committee of the University of Jyväskylä. All subjects gave written informed consent in accordance with the Declaration of Helsinki.

### Movement Actuator

**Figure 1:**
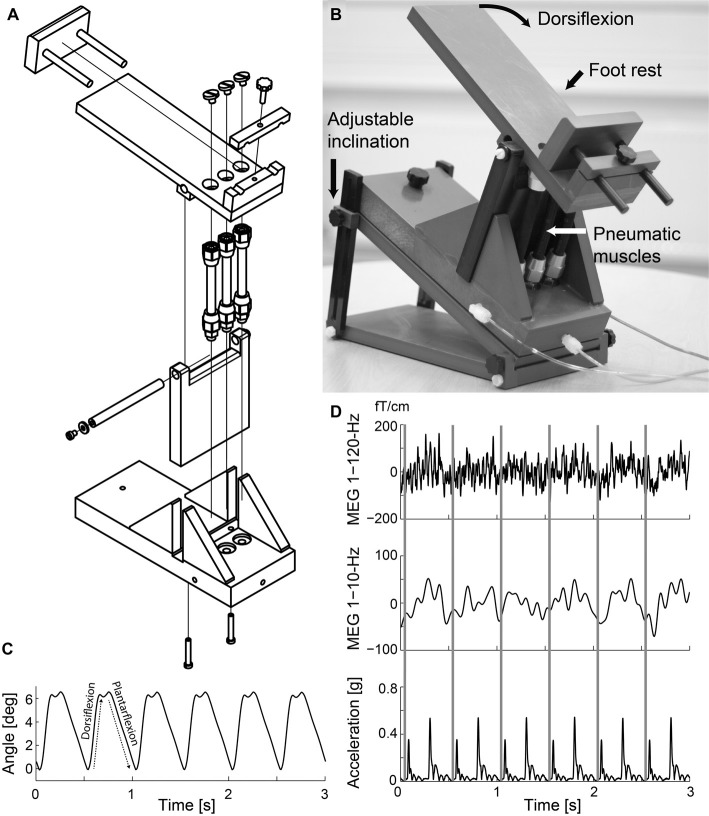
Ankle-movement actuator and representative signals. **(A)** Technical drawing of the actuator. **(B)** Individual’s right foot rested on the foot rest while repetitive ankle flexions were generated. **(C)** Ankle-angle signal while the ankle-movement actuator operated at 2 Hz. **(D)** Representative time-locked magnetoencephalographic (MEG) and accelerometer signals as a function of time when the right ankle of Subject 1 was moved at 2 Hz. Rows from top to bottom 1–120-Hz and 1–10-Hz MEG (from the most responsive channel), and Euclidian norm of the three orthogonal acceleration signals. The gray vertical lines indicate the onsets of dorsiflexion.

Figure 1 shows the custom-made non-magnetic ankle-movement actuator. The principle of this stimulator is similar as in our previously built device to generate finger and toe movements (Piitulainen et al., 2015). A pneumatic system is embedded into a PVC-plastic frame designed to support the individual’s foot. Three pneumatic artificial muscles (DMSP-10-100 AM-CM, diameter 10 mm, length of the contracting part 100 mm; Festo AG & Co., Esslingen, Germany) were attached vertically to the lower plate of the frame and the upper plate where the individual’s foot was resting. The pneumatic artificial muscles moved in a vertical direction when its internal air pressure (1–7 bar) changed, and thus rotated the upper plate. The pressure was regulated by a solenoid valve (SY5220-6LOU-01F-Q, SMC Corporation, Tokyo, Japan) that was controlled by computer-generated trigger pulses. The solenoid valve was placed outside the magnetically shielded room and three 3.5-m non-elastic tubes (internal diameter 2.5 mm) conveyed the airflow to the pneumatic artificial muscles. The pneumatic artificial muscles were first shortened simultaneously by increasing the air pressure (opening of the valve), thereby dorsiflexing the ankle joint, and then returned back to the initial position (plantarflexion of the ankle joint) when the air pressure was released (closing of the valve).

### Experimental Protocol

During MEG recordings, the subjects were sitting their eyes open with the stimulated leg on the movement actuator placed on the floor in front of them. The other leg was resting on the floor. The tested foot was attached to the actuator with an elastic strap to ensure its stability. Earplugs were used and Brownian noise was played in the background via a flat-panel speaker (Panphonics 60 × 60 SSHP, Tampere, Finland) to minimize concomitant auditory noise that arose from the movement actuator. A white A3-sized paper sheet, taped vertically to the MEG gantry, prevented the subjects from seeing the moving leg. Subjects were instructed to fixate on a black dot on the wall of the magnetically shielded room, 3 m in front of them. Brisk continuous dorsi-plantarflexion movement (movement range 6.6°, peak angular velocity 80°/s) was generated for the ankle joint at 2 Hz for 4 min in each leg separately. The stimulation order of the legs was randomized for each individual. Four minutes resting state data were collected similarly while no movements were generated.

On a separate day (1–4 days after the MEG session), postural stability was measured while subjects were standing quietly, unshod on a force plate (AMTI, OR6-6 model, Watertown, MA, USA) with either eyes open or eyes closed for 2 min (continuously) in each task. Typically a minimum of 90 s is required for reliable bipedal postural sway recordings (Ruhe et al., 2010). The subjects held their hands together in front at hip level and were asked to stand as still as possible. Feet were placed hip-width apart with toes facing directly forward. During the eyes-open task, the subjects gaze was fixated on a black dot at eye level 3 m in front. After a short break (<1 min), during which time the subject’s feet remained in the same position, the second task was performed.

### Measurements

#### MEG

The measurements were carried out at the Jyväskylä Centre for Interdisciplinary Brain Research. MEG signals were recorded in a magnetically shielded room (Magnetical Shielding Cabin, VACOSHIELD, Vacuumschmelze GmbH & Co. KG, Hanau, Germany) with a 306-channel whole-scalp neuromagnetometer (Elekta Neuromag^®^ TRIUX™, Elekta Oy, Helsinki, Finland). The recording passband was 0.1–330 Hz and the signals were sampled at 1 kHz. The individual’s head position inside the MEG helmet was continuously monitored by feeding current to five head-tracking coils. The coils were attached on the scalp prior to measurement and their locations were determined with respect to anatomical fiducials with an electromagnetic tracker (Fastrak, Polhemus, Colchester, VT, USA).

#### Acceleration and Trigger Signals

Hallux acceleration was recorded with a 3-axis accelerometer (ADXL335 iMEMS Accelerometer, Analog Devices Inc., Norwood, MA, USA) attached on the skin over the metatarsal bone. Acceleration was low-pass filtered at 330 Hz and sampled at 1 kHz, time-locked to MEG signals. Stability of the peak acceleration magnitude was quantified as the coefficient of variation for the peak value of the Euclidian norm of the three orthogonal accelerometer signals across all dorsiflexions separately for each individual and for left and right legs.

### Data Processing

#### Preprocessing

Continuous MEG data were first preprocessed off-line using temporal signal-space-separation with head movement compensation to suppress external interferences and to correct for head movements (Taulu and Simola, 2006). The MEG and acceleration signals were band-pass filtered offline at 0.4–195 Hz.

#### Coherence and Power Analysis

For coherence analyses, the continuous data were split into 2-s epochs with 1.6-s epoch overlap, leading to a frequency resolution of 0.5 Hz (Bortel and Sovka, 2007). MEG epochs with magnetometer signals >3 pT or gradiometer signals >0.7 pT/cm were excluded to avoid contamination by eye movements and blinks, muscle activity, or external MEG artifacts. We then performed coherence analysis (Halliday et al., 1995)—yielding cross-, power- and coherence-spectra, as well as cross-correlograms—between MEG signals and the Euclidian norm of the three orthogonal accelerometer signals. Before the coherence analysis, each epoch of acceleration was normalized by its Euclidian norm (Bourguignon et al., 2011). Peak CKC strength was quantified as the strongest coherence value across all MEG gradiometers at the movement frequency (F0; 2 Hz) and its first harmonic (F1; 4 Hz) separately from the respective coherence-spectra.

To study the possible effect of background-MEG power on CKC strength, the power spectra were computed for resting-state-MEG recording for the same gradiometer that showed the peak CKC value at F0 and F1. This power analysis was otherwise identical to the coherence analysis, but flat-top-weighted windows were used to allow power comparisons across the subjects. From the power spectra, power values for F0 and F1 were extracted and were further analyzed.

#### Cortical Movement-Evoked Fields

MEG signals were averaged with respect to the movement onsets of the ankle dorsiflexions to compute peak-to-peak-amplitude of the sustained-movement-evoked field for both legs, i.e., the strength of cortical activity related to the continuous 2-Hz movement.

#### Cortical Source of CKC

Cortical source for CKC was approximated from spatial distribution of cross-correlograms and the corresponding magnetic field patterns. No further detailed source localization was performed, since subjects’ anatomical MRIs were not available. The cross-correlograms were first band-pass filtered at 1–40 Hz and the source analysis was performed in the time domain, on the spatial distribution of the filtered cross-correlogram, as previously done in CKC studies (Bourguignon et al., 2011, 2013; Piitulainen et al., 2013b). Equivalent current dipole was estimated within the spherical head model at the most prominent peak of the filtered cross-correlogram, using a fixed selection of 84 sensors (52 gradiometers and 26 magnetometers) over the vertex. The most prominent peak of the cross-correlogram depicts the time-point of strongest coherence between two signals.

#### Postural Stability

Anterior-posterior (x) and medio-lateral (y) force signals of the force plate were sampled at 1000 Hz and low-pass filtered offline at 20 Hz. Postural sway was quantified by first computing xy-magnitude of center of pressure (COP) distance (mm) from sample-to-sample, and then multiplying it with the sampling frequency (1000 Hz) to obtain the mean velocity (mm/s) of the COP for both tasks separately. This measure combines both mediolateral and anteroposterior postural sway, and thus provides a measure to estimate overall postural stability performance. The effect of vision for the postural sway was quantified by subtracting the postural sway of the eyes-open task from the eyes-closed task, i.e., indicating the increase in the postural sway due to closing the eyes.

### Statistical Analysis

All statistical analyses were performed in IBM SPSS Statistics software (ver. 24). Data was first ensured to have normal distribution using the Shapiro-Wilk test. Independent-samples *t*-test was used in between-group (younger vs. older) comparisons for CKC strength, number of averages, sustained field peak-to-peak amplitude, acceleration magnitude and postural sway. Paired-samples *t*-test was used to compare CKC strength, number of averages, sustained field peak-to-peak amplitude, MEG power and acceleration magnitude for dominant and non-dominant legs, and to compare postural sway between eyes-open and eyes-closed tasks. Pearson correlation coefficient was computed across all subjects between CKC strength, and MEG response amplitude and background-MEG power to estimate the effect of the MEG signal strength to the CKC strength. Linear multiple regression analysis was performed to estimate the importance of CKC strength (for non- and dominant legs and F0 and F1 separately), body mass, height, gender, age, group (younger, older) on predicting postural stability (during eyes open and closed conditions, and their difference) using the enter method in SPSS. Results are indicated as mean ± standard deviation.

## Results

Figure 1 illustrates the ankle-movement actuator, and MEG and acceleration signals of a representative individual (Subject 1) during the continuous 2-Hz movement. The actuator did not produce notable artifacts in the MEG signals, and thus the strong fluctuations at the movement frequency reflect the cortical processing of proprioceptive afference. The acceleration signals contained two clear peaks for each movement cycle, reflecting the initial phases of the dorsi- and plantarflexions. Peak acceleration magnitude (for dorsiflexion) was 3.0 ± 0.36 m/s^2^ for the dominant foot (coefficient of variation across movements 6.4 ± 4.7%) and 3.1 ± 0.46 m/s^2^ for the non-dominant foot (coefficient of variation 5.0 ± 2.4%). There were also no differences in the peak acceleration magnitude between feet (*p* = 0.548) or groups (younger 3.12 ± 0.16 m/s^2^ vs. older 3.08 ± 0.35 m/s^2^, *p* = 0.806).

No significant differences were observed in body mass (*p* = 0.579), height (*p* = 0.422) or habitational level of physical activity (*p* = 0.874) between the groups.

### Corticokinematic Coherence

#### Younger vs. Older

**Figure 2:**
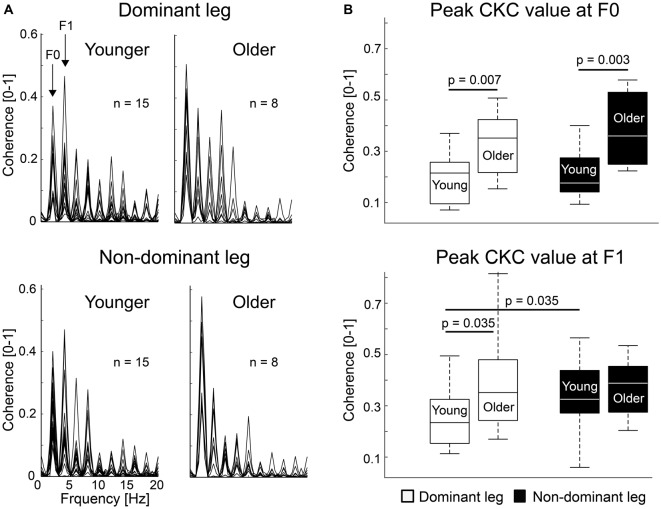
Individual corticokinematic coherence (CKC) spectra and group averages. **(A)** CKC spectra showed clear peaks at movement frequency (F0) and its first harmonic (F1) for dominant and non-dominant ankle movements. Maximum coherence between the acceleration signal and MEG signal of the sensor showing the peak CKC at F0 is shown. **(B)** CKC at F0 and F1 for dominant and non-dominant legs in younger and older groups. Error bars indicate range. Vertical line indicates median. Horizontal boundaries of the boxes indicate quartiles.

Figure 2A shows the MEG–acceleration coherence spectra superimposed for all subjects. Coherence showed a clear peak in all subjects and both legs at the movement frequency (F0) and its first harmonic (F1). Due to the regular movement, several harmonics are visible.

Figure 2B shows peak CKC strength for dominant and non-dominant legs at F0 and F1. CKC ranged from 0.06 to 0.81 (mean ± SD, F0: 0.26 ± 0.13; F1: 0.32 ± 0.15), peaking at the gradiometers over the vertex. At F0, CKC was ~76% stronger for the older than younger subjects when the legs were pooled together (older 0.36 ± 0.14 vs. younger 0.20 ± 0.09, *p* = 0.001). CKC was stronger in older than in younger subjects for the dominant (~69%, older 0.33 ± 0.13 vs. younger 0.20 ± 0.09, *p* = 0.007) and non-dominant (~83%, older 0.38 ± 0.15 vs. younger 0.21 ± 0.09, *p* = 0.003) legs separately as well. At F1, CKC was not significantly different between the groups when the legs were pooled together (older 0.38 ± 0.15 vs. younger 0.29 ± 0.10, *p* = 0.077) or for the non-dominant leg (older 0.37 ± 0.12 vs. younger 0.33 ± 0.13, *p* = 0.433). However, CKC was ~60% stronger for the older than younger subjects for the dominant leg (older 0.39 ± 0.21 vs. younger 0.25 ± 0.11, *p* = 0.035).

#### Dominant vs. Non-dominant Leg

At F0, CKC strength did not differ between the legs in younger (dominant 0.20 ± 0.09 vs. non-dominant 0.21 ± 0.09, *p* = 0.650) or older (dominant 0.33 ± 0.13 vs. non-dominant 0.38 ± 0.15, *p* = 0.310) subjects, or when the groups were pooled together (dominant 0.24 ± 0.12 vs. non-dominant 0.27 ± 0.14, *n* = 23, *p* = 0.263). However, at F1, in the younger subjects, CKC was stronger (*p* = 0.037) in the non-dominant (0.33 ± 0.13) than dominant leg (0.25 ± 0.11).

### Number of Epochs, Response Amplitude and Background-MEG Power

The number of epochs in the coherence analysis did not differ between the legs (dominant: 560 ± 74 vs. non-dominant: 572 ± 40, *p* = 0.411) or groups (younger: 574 ± 46 vs. older: 551 ± 51, *p* = 0.275).

Sustained field peak-to-peak amplitude generated by the 2-Hz movement did not differ between the legs (dominant 24.4 ± 8.0 fT/cm vs. non-dominant 23.9 ± 6.8 fT/cm, *p* = 0.784) or groups (younger 24.1 ± 5.6 fT/cm vs. older 24.2 ± 6.1 fT/cm, *p* = 0.958). Similarly, no differences were observed in the background-MEG power between the legs at F0 (dominant 131 ± 77 fT/cm^2/Hz^ vs. non-dominant 131 ± 88 fT/cm^2/Hz^, *p* = 0.983) and at F1 (dominant 71 ± 36 fT/cm^2/Hz^ vs. non-dominant 70 ± 36 fT/cm^2/Hz^, *p* = 0.849), or the groups at F0 (younger 146 ± 85 fT/cm^2/Hz^ vs. older 103 ± 43 fT/cm^2/Hz^, *p* = 0.361) and at F1 (younger 73 ± 40 fT/cm^2/Hz^ vs. older 65 ± 28 fT/cm^2/Hz^, *p* = 0.980). The sustained field peak-to-peak amplitude or background-MEG power did not correlate with CKC strength or postural stability.

### Cortical Source of CKC

**Figure 3:**
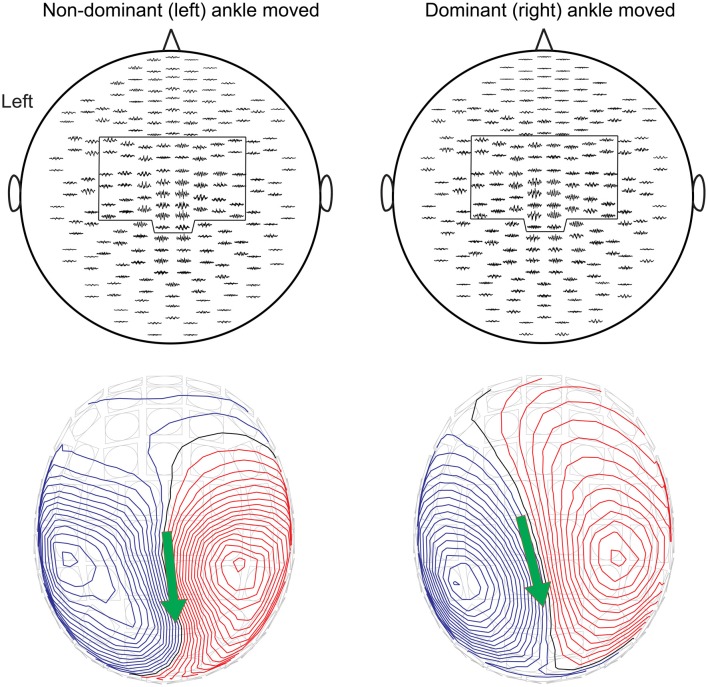
The cross-correlograms for all gradiometers and the corresponding magnetic field patterns superimposed on the MEG sensor array for Subject 1 during non-dominant (left panels) and dominant (right panels) ankle movements at 2 Hz. The pre-selected subsets of sensors are outlined. Magnetic field patterns were obtained from Equivalent current dipole estimation at the main peak of the cross-correlogram. The red isocontour lines indicate the flux out of the skull and the blue lines flux into the skull. The arrow depicts the surface projection of the equivalent current dipoles orientation.

Figure 3 shows the cross-correlograms for all gradiometers of the MEG sensor array and the respective magnetic field patterns for Subject 1. Equivalent current dipole was estimated at the time of the most prominent peak of the cross-correlogram, and showed clear dipolar field patterns centered on interhemispheric sensors near the central sulcus. Similar patterns were observed in all subjects. The applied fixed selections of MEG sensors are outlined.

### Postural Stability

**Figure 4:**
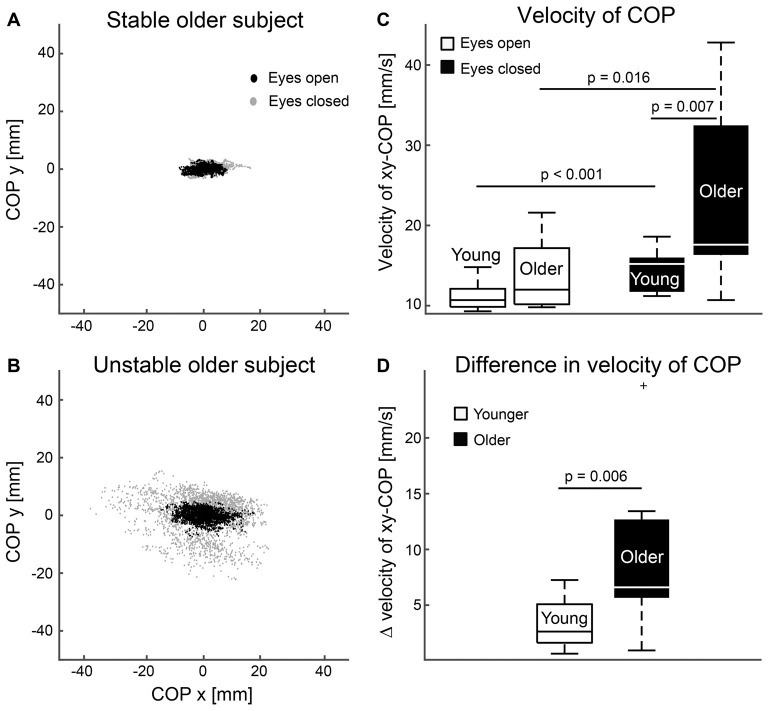
Postural stability. **(A,B)** Superimposed center of pressure (COP) distributions during standing eyes open (black dots) and closed (gray dots) for one stable **(A)** and unstable **(B)** older subject. **(C)** Magnitude of postural sway for eyes open and closed tasks. **(D)** Difference in mean magnitudes of postural sway between eyes closed and open tasks. Error bars indicate range. Vertical line indicates median. Horizontal boundaries of the boxes indicate quartiles.

Figure 4 shows superimposed COP distributions during standing with eyes open and closed for two older subjects with clearly different balance performance. COP distributions increased especially in the older subjects when they closed their eyes. On average, postural sway was higher during the eyes-closed task for younger (eyes open 11.14 ± 1.74 mm/s vs. eyes closed 14.32 ± 2.43 mm/s, *p* < 0.001) and older (eyes open 13.82 ± 4.53 mm/s vs. eyes closed 23.48 ± 11.56 mm/s, *p* = 0.016) subjects. Between-group comparisons showed that postural sway was ~64% higher for the older than younger subjects while standing with eyes closed (*p* = 0.007), and the sway increased more from the eyes-open to eyes-closed task in older than younger subjects (~29% in younger 3.18 ± 2.09 mm/s vs. ~70% in older 9.66 ± 7.71 mm/s, *p* = 0.006). During eyes-open standing, postural sway was ~24% higher for the older than younger subjects, but did not reach the level of statistical significance (*p* = 0.055).

### Predicting Postural Stability

Using the enter method, a significant regression models emerged for postural stability explaining 68% (adjusted R square = 0.68) of postural sway during eyes-open (*F*_(9,12)_ = 2.89, *p* = 0.045) and 72% during eyes-closed (*F*_(9,12)_ = 6.97, *p* = 0.001) condition, and 71% of their difference (*F*_(9,12)_ = 6.66, *p* = 0.002). Table 1 presents standardized Beta coefficients and *p*-values for each predictor variable of the linear regression models. Significant variables were CKC at F1 for dominant leg (eyes open: beta 0.722, *p* = 0.011; eyes closed: beta 0.911, *p* < 0.001; difference: beta 0.903, *p* < 0.001) and age (only for difference: beta 2.371, *p* = 0.036). Body mass, height, gender, group and CKC at F0 or at F1 in non-dominant leg were not significant predictors in these models.

**Table 1:** Beta coefficients from linear regression model to predict postural stability.

	COP eyes open		COP eyes closed		COP difference	
Predictor variable	Beta	*p*-value	Beta	*p*-value	Beta	*p*-value
Weight	−0.240	0.401	0.064	0.751	0.229	0.276
Height	0.480	0.176	0.253	0.307	0.093	0.709
Age	−0.544	0.701	1.429	0.173	2.371*	0.036*
Group	0.636	0.645	1.092	0.278	1.937	0.071
Gender	0.484	0.247	0.523	0.090	0.479	0.123
CKC F0 dominant	−0.186	0.525	−0.098	0.637	−0.036	0.866
CKC F0 non-dominant	0.660	0.052	0.281	0.222	0.030	0.895
CKC F1 dominant	0.722*	0.011*	0.911***	0.000***	0.903***	0.000***
CKC F1 non-dominant	0.244	0.353	−0.155	0.404	−0.363	0.071

*p < 0.05, ***p < 0.001.

## Discussion

Our results indicated that cortical proprioceptive processing is affected by aging. Older subjects showed stronger CKC for ankle movements (contrary to our hypothesis) and higher postural sway in line with previous evidence (Hasselkus and Shambes, 1975; Era and Heikkinen, 1985; Baloh et al., 1994). The degree of postural sway was predicted by CKC strength, and also with subjects’ age when the postural task shifted from eyes-open to eyes-closed condition, i.e., when relying primarily on proprioception. The differences in CKC and postural sway may be explained by age-related motor deterioration, especially since the groups did not differ in habitual physical activity levels. Little is known about age-related proprioceptive deterioration, especially at the cortical level, however cortical control of upright stance seems to be altered due to impaired somatosensory processing in older subjects (Ozdemir et al., 2018). CKC could potentially be used as a tool to unravel this topic, and even as a “biomarker” for motor and somatosensory impairments. Indeed, stronger CKC seems to suggest lower motor performance, skill or level of usage, as the non-dominant leg showed stronger CKC among the younger subjects. Finally, the novel ankle-movement actuator led to a strong coherence between kinematics and cortical MEG signals, in accordance with earlier findings of finger and toe movements (Piitulainen et al., 2015).

### CKC in Younger and Older

We have shown previously that CKC primarily reflects proprioceptive processing in the SM1 cortex with negligible effect of cutaneous input when measured with MEG (Piitulainen et al., 2013a; Bourguignon et al., 2015), and thus CKC has been assumed to quantify cortical proprioceptive processing. However, it has been unclear whether stronger CKC indicates more precise and/or stronger coupling between proprioceptors and cortical activity. Nevertheless, we expected stronger CKC in the younger subjects, because of their better proprioceptive perception capabilities (joint position sense, see Skinner et al., 1984; Kaplan et al., 1985). Our current results indicated the opposite dependency, and consequently our hypothesis should be rejected. CKC was stronger in the older subjects who also showed more postural sway during eyes-closed standing. This task relies strongly on proprioception (Fitzpatrick and McCloskey, 1994). It, thus, appears that CKC strength does not solely reflect the “amount” of proprioceptive afference, but reflects some other aspects of cortical proprioceptive processing.

It is difficult to define a conclusive neuronal mechanism for the strong CKC in the older subjects, but it is clear that the proprioceptive afference activated the cortex differently in the older subjects. Based on simulations, an increase in signal amplitude may increase the level of coherence (Muthukumaraswamy and Singh, 2011). However, in the present study, the sustained-field amplitudes were similar in younger and older subjects. Therefore, the strong CKC was not explained by amplitude differences in MEG or acceleration signals. Furthermore, the clear differences in CKC strength were not affected by background-MEG power. The younger and older subjects showed similar levels of background-MEG power during resting state recording at both F0 (at 2 Hz) and F1 (at 4 Hz), and the background-MEG power did not correlate with CKC strength.

As cortical activation was not stronger in the older vs. younger subjects, the proprioceptive processing has to be intrinsically different. Indeed, older subjects do show stronger modulation at 30–50-Hz EEG signals to changing sensory conditions (e.g., closing the eyes) during upright stance than younger subjects (Ozdemir et al., 2018), possibly due to impaired proprioceptive processing in the older subjects. In addition, both voluntary-movement-related cortical potentials (Kita et al., 2001; Wright et al., 2012a) and BOLD-fMRI response (Jäncke et al., 2000; Krings et al., 2000) have shown to be reduced in the sensorimotor cortex of motor-skilled subjects, and to decline during motor-skill training (Wright et al., 2012b). This suggest that lesser neurons (i.e., more distinct neuronal network) need to be recruited in the skilled-motor system enabling more precise and efficient neuronal processing. In contrast to the aforementioned voluntary movements, proprioceptive stimulation (tendon vibration) has shown to produce stronger percent-signal change in BOLD-fMRI for subjects with better balance performance in various cortical and subcortical structures—however—not in the SM1 cortex (Goble et al., 2011).

We suggest, that the proprioceptive afference may be processed in a more specific manner in the cortex—likely involving a smaller neuronal population—of the younger subjects with more precise balance performance. This suggestion was further supported by the observed stronger CKC for the non-dominant than dominant leg at F1. However, this was significant only among the younger subjects. Furthermore, strong CKC at F1 in the dominant leg seems to predict worse postural balance. Therefore, strong CKC possibly reflects inefficient cortical processing of the proprioceptive afference, and/or functional deficits in the peripheral proprioceptors and spinal circuits. It may be that the stronger CKC in the older subjects reflects an attempt to compensate and overcome these potential proprioceptive deficits. However, future studies are needed to confirm this suggestion with larger sample sizes and longitudinal designs including various motor tasks for both upper and lower limbs.

Aging-related sensorimotor deterioration is the most potential explanation for the current results. Gray matter thickness is significantly diminished during aging (Magnotta et al., 1999; Sowell et al., 2003), and more dense gray matter predicts better postural stability both in younger and older adults (Boisgontier et al., 2016). Atrophy of the muscle begins at ~25 years (Lexell et al., 1988) of age and is eventually associated with loss of spinal motoneurons innervating the muscle (Rowan et al., 2012; Hepple and Rice, 2016). Equivalently, it may be that sensorimotor afferents are also lost with aging, which may explain the inefficient cortical proprioceptive processing. Indeed, breakdown of white matter tracts occurs with aging (Bartzokis et al., 2004; Kochunov et al., 2007, 2009), but, for example, this is less evident among early developed thickly myelinated commissural fibers of mid-body of corpus callosum conveying motor and proprioceptive information than in later developed smaller fibers in the genu of the corpus callosum (Kochunov et al., 2007). Nevertheless, aging clearly modifies the function of the spinal and corticospinal sensorimotor circuits. For example, corticospinal excitability is increased and spinal excitability is decreased with aging when quantified with motor-evoked potentials to transcranial magnetic stimulation and Hofmann’s reflex, respectively (Baudry et al., 2015). Consequently, CKC could potentially be used as tool to unravel these mechanisms at the cortical level, and even detect cortical proprioceptive impairments.

### CKC and Postural Stability

This is the first study to suggest that CKC strength may predict postural stability performance. This finding further strengthens the view that CKC primarily reflects cortical proprioceptive processing, which played a crucial role in the currently tested functional tasks. However, the direction of the association was unexpected. Subjects with better postural stability showed weaker CKC and vice versa. Interestingly, three (out of seven) older subjects who showed the most unstable postural balance had also the strongest CKC. However, future studies with larger sample sizes are needed to confirm the importance and causality of this observation.

Our results support previous findings on aging and proprioception. Proprioceptive perception (joint position sense) is impaired with aging (Skinner et al., 1984; Kaplan et al., 1985), which is reflected by increased postural sway in older adults during standing (Hasselkus and Shambes, 1975; Era and Heikkinen, 1985)—both for eyes-open and eyes-closed tasks (Baloh et al., 1994). Thus, both postural sway (Fitzpatrick and McCloskey, 1994) and CKC (Piitulainen et al., 2013a; Bourguignon et al., 2015) are strongly related to proprioception. Furthermore, older individuals have shown stronger modulation at 30–50-Hz EEG signals to changing sensory conditions (e.g., closing the eyes) during upright stance than younger individuals (Ozdemir et al., 2018), i.e., cortical activity related to control of upright stance seems to be altered with aging. Based on aforementioned findings, our results are likely explained by age-related proprioceptive deterioration.

In case of corticomuscular coherence (between MEG/EEG and electromyography), abnormally high coherence has been observed in impaired motor system, e.g., in Parkinson’s disease patients manifesting cortical myoclonus (Caviness et al., 2003). However, corticomuscular coherence reflects efferent common synaptic inputs from cortex to spinal motoneurons at ~20 Hz (Conway et al., 1995; Baker et al., 1997; Salenius et al., 1997), whereas CKC reflects proprioceptive afference at the movement frequency (<3 Hz for voluntary movements). A recent study shows that effective cortical proprioceptive processing operates at <3 Hz frequencies—not at ~20 Hz—even during steady isometric contractions (Bourguignon et al., 2017). Therefore, the corticomuscular coherence and CKC are not comparable phenomena, although both phenomenon can provide their specific insights for understanding the diseased and aging motor system.

### Cortical Sources

The CKC peaked on the MEG sensors over the vertex, around the expected foot area of the SM1 cortex, in good accordance with CKC for toe movements (Piitulainen et al., 2015). Individuals’ anatomical MRIs were not available, and thus we could not confirm the exact anatomical location of the coherent source. Nevertheless, the corresponding magnetic field patterns obtained at the main peak of the cross-correlograms were adequately explained by a single equivalent current dipole. These results indicate that the ankle-movement actuator can also be used in functional mapping of the SM1 cortex, either alone or as part of a multimodal functional mapping scheme (Bourguignon et al., 2013).

### Benefits and Limitations of the Ankle-Movement Actuator

The ankle-movement actuator did not produce distinct mechanical or electric artifacts into the MEG signals, and it created only subtle acoustic noise that were well masked with earplugs and background sound (Brownian noise). The kinematics of the successive ankle rotations were stable. The actuator can thus be used as a tool to quantify proprioceptive processing in the cortex, and also to locate the human SM1 cortex for the foot.

Computer-controlled movement actuator can achieve reproducible millisecond level accuracy of the movements (Xiang et al., 1997a,b; Druschky et al., 2003; Woldag et al., 2003; Muthukumaraswamy, 2010; Onishi et al., 2013; Piitulainen et al., 2013a), which is crucial for longitudinal studies of for example stroke recovery or motor learning. CKC is recorded using continuous movements that can be produced using pneumatic-artificial muscles, but is more difficult with pneumatic-cylinder-based devices (Lange et al., 2001; Alary et al., 2002).

A limitation of the pneumatic-artificial muscle is that only movements up to ~20 Hz can be generated, due to incomplete release of the air pressure from the pneumatic muscle at high stimulation rates. Thus, movement range is reduced with movement frequency, but is dependent also on the length of the pneumatic muscle, which can contract up to 25% of its resting length. Ten centimeter long pneumatic-artificial muscles were used in the current actuator. In order to maximize the movement velocity, they had to be attached close to the rotation axis of the foot rest, leading to suboptimal lever arm for their force generation. Thus, if one aims to use active tasks—e.g., active resistive plantarflexion during the movement stimulation—the design of the actuator needs to be modified.

We used a passive task to stimulate primarily the proprioceptors of the ankle joint. It is impossible to completely block the effect of cutaneous stimulation of the sole during passive-ankle rotations. However, our previous results indicate that cutaneous stimuli do not have significant effect on the strength of CKC during either active or passive index-finger movements (Piitulainen et al., 2013a). Nevertheless, it is important to recognize that passive movements always activate both proprioceptors and cutaneous receptors—both providing important information for the central nervous system, e.g., during human locomotion.

### Future Prospects and Limitations

Although both CKC and age were significant predictors for postural sway, the sample size in the current study was small and unequal between younger and older subjects. Hence, the study design was not planned to confirm causalities between CKC and postural sway. Therefore, future studies with substantially larger sample sizes and longitudinal designs are needed to confirm the possible associations and causalities between CKC strength and functional motor performance throughout the lifespan.

It still needs to be confirmed how CKC correlates with postural stability and other types of motor skills, e.g., with fine-motor skills of the hand. This would be important to confirm, as CKC could potentially be used to detect and evaluate the effect of impairment in cortical proprioceptive processing during aging and in various motor disorders. The advantage of CKC is that it performs well even in patients with strong magnetic artifacts in MEG signals, e.g., due to cranial clips et cetera (Bourguignon et al., 2016), and can be performed even in newborns (Smeds et al., 2017). Due to high stability in the elicited passive movements, CKC is an especially suitable tool for longitudinal studies to follow changes in efficiency of cortical proprioceptive processing during development, aging, training, rehabilitation, motor-skill acquisition, etc.

## Conclusion

Our results imply that cortical proprioceptive processing is hindered with aging. Stronger CKC and higher postural sway was detected in older than younger subjects. Aging-related motor deterioration may thus hinder cortical proprioceptive processing, that may also be less efficient in the non-dominant leg. CKC does not seem to solely depend on the amount of proprioceptive afference, but may reflect precision and efficiency of the cortical proprioceptive processing. Our novel ankle-movement actuator can be used to examine cortical proprioceptive processing using the CKC, and has the potential to unravel proprioception-related-neuronal mechanisms both in healthy and disease to study aging, balance, motor-skill acquisition, rehabilitation, motor-disorder etiology, etc.

## Author Contributions

HP, SW and JA designed research. HP, SS and TP piloted the MEG design. SS did MEG recordings. SW did balance measurements. HP analyzed data. HP, SW, TP and JA wrote the manuscript.

## Conflict of Interest Statement

The authors declare that the research was conducted in the absence of any commercial or financial relationships that could be construed as a potential conflict of interest.

## Abbreviations

CKC: corticokinematic coherence
COP: center of pressure
EEG: electroencephalography
F0: movement frequency
F1: first harmonic of the movement frequency
fMRI: functional magnetic resonance imaging
MEG: magnetoencephalography
SM1: primary sensorimotor.
